# Implementation research evidence uptake and use for policy-making

**DOI:** 10.1186/1478-4505-10-20

**Published:** 2012-07-02

**Authors:** Ulysses Panisset, Tracey Pérez Koehlmoos, Ahmad Hamdi Alkhatib, Tomás Pantoja, Prabal Singh, Jane Kengey-Kayondo, Ben McCutchen, Miguel Ángel González Block

**Affiliations:** 1 Coordinator, Evidence Informed Policy Network (EVIPNet), Department of Knowledge Management and Sharing, WHO, Avenue Appia 20, 1211, Geneva 27, Switzerland; 2 Programme Head, Health & Family Planning Systems Programme, ICDDR,B, 68 ShahedTajuddin Ahmed, Sarani, Mohakhali, Dhaka, 1212, Bangladesh; 3 Faculty of Health Sciences, McMaster University; Forum Fellow, McMaster Health Forum, 1280 Main St. West, Hamilton, ON, L8S 4L6, Canada; 4 Family Medicine Department, Pontificia Universidad Católica de Chile, Avenue Libertador Bernardo O’Higgins 340, Santiago, Chile; 5 ACCESS Health International Inc., Centre for Emerging Markets Solutions, Indian School of Business, Gachibowli, Hyderabad, 500 032, Andhra Pradesh, India; 6 Strategic Alliances, Special Programme for Research and Training in Tropical Diseases (TDR), WHO, 20 Avenue Appia, CH-1211, Geneva 27, Switzerland; 7 Faculty of Health Sciences, McMaster University, 1280 Main St. West, Hamilton, ON, L8S 4L6, Canada; 8 Centre for Health Systems Research Institute, Av. Universidad 655 Col. Santa María Ahuacatitlán Cuernavaca, (CP 62100), Morelos, México

**Keywords:** Evidence-informed policy, Implementation, Knowledge translation, Policy, Policy-making

## Abstract

A major obstacle to the progress of the Millennium Development Goals has been the inability of health systems in many low- and middle-income countries to effectively implement evidence-informed interventions. This article discusses the relationships between implementation research and knowledge translation and identifies the role of implementation research in the design and execution of evidence-informed policy. After a discussion of the benefits and synergies needed to translate implementation research into action, the article discusses how implementation research can be used along the entire continuum of the use of evidence to inform policy. It provides specific examples of the use of implementation research in national level programmes by looking at the scale up of zinc for the treatment of childhood diarrhoea in Bangladesh and the scaling up of malaria treatment in Burkina Faso. A number of tested strategies to support the transfer of implementation research results into policy-making are provided to help meet the standards that are increasingly expected from evidence-informed policy-making practices.

## Introduction

The 2010 Millennium Development Goals (MDGs) Report reveals that without a major push forward, many of the MDG targets are likely to be missed in most regions [1]. A major obstacle to the progress of the MDGs has been the inability of health systems in many low- and middle-income countries to effectively implement evidence-informed interventions. Consider MDG 6 (combat HIV/AIDS, malaria and other diseases), which explicitly outlines that people at risk of malaria (and especially children less than 5) should be sleeping under insecticide-treated bed nets. The value of insecticide-treated bed nets is supported by high quality systematic reviews, which indicate that bed nets are highly effective in reducing childhood mortality and morbidity from malaria [2]. However, only 35% of young children were sleeping under bed nets in 2010, still below the World Health Assembly target of 80% [3]. In general, nearly 14,000 people in sub-Saharan Africa and South Asia die daily from preventable and/or treatable diseases that in some cases have been eliminated in developed countries, including malaria, HIV and diarrheal diseases [4].

This illustrates that the availability of evidence on the effectiveness of an intervention is necessary, but not sufficient to produce better health outcomes. Barriers and facilitators of implementation must be identified and strategies must be designed in order to improve the chances of successfully delivering a proven intervention. Implementation research can detect barriers and facilitators of delivery and characterize strategies that can address and utilize, respectively, such factors [5,6]. However, generating implementation research evidence is not sufficient to improve the chances of successfully delivering an intervention; it is also important that innovative tools are used to ensure that implementation research is available for uptake and use by policy-makers. As international agencies are increasingly recognizing, “research helps us to identify what works, what does not work and how to understand the local context when introducing new ways of working [but] there is no point doing research if the findings do not get into policy and practice” [7]. Therefore, emphasis must be placed not only on the production of implementation research, but also on its integration into knowledge translation (KT) processes to ensure its use by policy-makers and programme managers.

KT is defined by the Canadian Institutes for Health Research (CIHR) as “…a dynamic and iterative process that includes synthesis, dissemination, exchange and ethically-sound application of knowledge, through sustainable partnerships to improve the health of citizens, provide more effective health services and products and strengthen the health care system” [8]. The main objective of this paper is to explore the different ways in which implementation research can inform policy-making through KT processes as they are integrated into knowledge translation platforms [9]. To this end, the article discusses the relationships between implementation research and KT and identifies the role of implementation research in the design and implementation of evidence-informed policy.

## Benefits of using implementation research in knowledge translation to inform policy-making

Many promising health interventions, when implemented, fail to achieve their maximum potential and/or reach those most in need. In some cases they may even cause economic or health harms. Health systems interventions are often social experiments; there is the potential for not only positive, but also null or negative outcomes [10,11]. If policy-makers are unaware of, or ignore evidence on the root causes of problems in the implementation of interventions, they increase the chances of producing negative outcomes. This includes wasting precious resources on inadequately designed programmes and policies and, more importantly, causing harm to human lives. Implementation research “aims to develop strategies for available or new health interventions in order to improve access to and use of these interventions by the populations in need” [6]. By identifying the barriers and facilitators and trying to address them in the design of strategies, implementation research increases the chances of producing positive outcomes.

It is well established that implementation research can be used to improve access to vaccines, treatments, diagnostics, and interventions. However, implementation research also can be used to strengthen health systems, improve patient safety, expand community based interventions and strengthen public-private partnerships [12]. Political support and interdisciplinary frameworks are still in need of developing in some of the newer areas that can employ of implementation research. To that end in 2011 a ‘road map for action’ was developed by World Health Organization’s Special Programme for Training and Research on Tropical Diseases [12].

Synergies between implementation research and KT improve demand, delivery and access to interventions. Implementation research can inform at multiple points in the policy-making process. Research can be used to identify problems, set country priorities during the design stages of policymaking, monitor and evaluate policies and programmes and/or ensure the successful implementation of policies once they have been devised. At each point, implementation research can address the political, historical, cultural, socioeconomic, health services and/or resource factors that affect policy-making and policy implementation. To summarize, implementation research can facilitate policy-making by helping to:

· Map the political and institutional context in which policies will be implemented,

· identify barriers to implementation and the determinants which prevent effective access to interventions,

· develop practical solutions and monitor and evaluate new implementation strategies,

· introduce evidence-informed implementation strategies in health systems,

· facilitate full scale implementation and

· collaborate in policy evaluation and modification*.*

KT can facilitate the use of implementation research in policy-making, helping politicians, policy-makers and managers to make better decisions. As discussed later in the article, KT processes aided policy-makers in Bangladesh in integrating implementation research in decision-making on the scale-up of zinc use for childhood diarrhea. The article also discusses the use of implementation research by KT platforms, such as the WHO’s Evidence Informed Policy Network (EVIPNet). KT platforms integrate KT processes, which are characterized by the following elements:

· A governing body comprising of representatives from groups of producers, purveyors and users of evidence,

· the political will to act based on the best available evidence,

· regular priority-setting processes to ensure that efforts to link research to action are highly relevant to the needs of potential research users,

· efforts to push evidence into policy-making in areas where actionable messages have been identified and

· a range of efforts to facilitate user pull of evidence [9].

## Challenges and opportunities for translating implementation research evidence

Implementation research faces technical and political challenges in informing policy when addressing the delivery of innovative programmes, services and/or tools. At this stage, substantial investments will have been committed towards the development of the intervention, putting stakeholders at great political risk. As such, a large number of stakeholders will favor the implementation of the policy, although they will not necessarily agree as to the feasibility of the expected benefits. The implementation of a policy may also face opposition at multiple decision points; the resistance may come from a programme manager concerned with costs and profit margin, the target population or healthcare providers. In either case, implementation research can provide evidence to either back or refute the basis for opposition or support. Stakeholders will therefore interpret evidence from implementation research through both technical and political contexts [13]. Further there are issues such as opposition from industry and potentially from other sectors as well as high turn-over among political appointees working in government agencies. For policy-makers in particular, evidence may be perceived as a way of reducing political and legal risk, as it allows politicians to acknowledge that evidence to inform decisions about public programmes is incomplete. In the case that programmes do not work as expected, research can also provide alternative courses for action [11].

The differences between policy-makers and researchers that have been exhaustively identified and described in the KT literature represent another challenge for the uptake of implementation research. For instance, in Uganda, there is evidence that implementation research results are valued differently by researchers and policy-makers. While researchers place importance on research evidence that is backed by rigorous methodology, policy-makers are more concerned about evidence that captures feasibility constraints.

KT offers opportunities to help overcome the challenges in the uptake and use of implementation research. KT tools and strategies can help researchers to deliver evidence that is packaged to address the concerns and needs of decision makers [5]. This includes packaging evidence to address dimensions of feasibility, including social acceptability, cost-effectiveness, community benefits and health system readiness to deliver the intervention [14]. Some of these KT processes include:

· Encouraging researchers to develop and translate implementation research that targets current health systems needs,

· packaging systematic reviews of relevant, wide-ranging and high quality implementation research literature focused on specific recommendations,

· preparing policy briefs and other executive summaries on implementation research results focused on policy makers and

· scientific publications with shared authorship between researchers and policy makers [5,15].

Interactive KT processes offer opportunities to overcome the technical and political barriers for the uptake of implementation research, as well as helping policy-makers and researchers to identify mutual interests, to relate to research evidence with greater trust and interest and to learn how to better work together. Systematic reviews have shown that “interactions between researchers and health care policy-makers increased the prospects for research use by policy-makers” [16]. Some of these interactive KT processes include:

· Preparing a deliberative policy dialogue (or other stakeholder-engagement approaches) as a key step to review and start implementing the policy options, with direct involvement of policy-makers, managers, health care providers and researchers to elicit tacit knowledge and evaluate feasibility,

· priority setting exercises whereby policy makers and researchers develop a shared implementation research and policy agenda,

· clearinghouses of easy-to-access and clearly relevant case studies, systematic reviews and other publications relevant for implementation of specific policies and packaged to facilitate its use in policy-making and

· learning workshops for decision makers with researchers to encourage collaboration on finding, appraising (both quality and relevance) and applying implementation research [5,17].

The deliberative dialogues can be especially designed to elicit policy-makers’ tacit knowledge and negotiating positions as a starting point to identify the research evidence to confirm or critique their positions [18]. The research results used to inform such dialogues must be thoroughly evaluated and graded to decide how much confidence to place in the evidence presented [19]. Interactive processes like deliberative dialogues also help develop a common value framework across researchers and other stakeholders, promoting team building and distributed leadership for action. Trust depends on the selection of stakeholders for dialogues. Participants must be carefully chosen and comply with house-rules on the diffusion of deliberations and on the confidentiality of participants [17].

Accurate reports in the mass media represent another important means of delivering up-to-date evidence to policy-makers. Researchers can work with journalists to use media reports as means of disseminating evidence to decision makers in a timely manner, while also informing the public. Several strategies and tools have been developed in order to facilitate the accurate reporting of research by journalists, including structured press releases, fact boxes, press conferences, providing ready-to-use stories, avoiding jargon in communication, providing access to experts, tip sheets and providing workshops and other types of training to help journalists gain a greater understanding of evidence-informed health policymaking [14,20]. Some researchers develop media releases for systematic reviews and profile and place in context locally conducted studies [9], providing evidence that is both timely and relevant.

## An example of implementation research supporting KT from Bangladesh

The scale up of zinc use for childhood diarrhea in Bangladesh, known as the ‘SUZY Project’ for Scaling Up Zinc for Young Children with Diarrhea, illustrates the use of KT strategies in encouraging the uptake of implementation research by policy-makers. Systematic reviews of the research literature and on a joint UNICEF/WHO recommendation established that zinc provides a very effective treatment for diarrhea among children under five years of age by reducing the severity and duration of diarrhea as well as the likelihood of future episodes of diarrhea and the need for hospitalization. It was estimated that zinc treatment could save the lives of 30,000 to 75,000 children per year in Bangladesh alone [21-25].

As a first step towards implementing this promising intervention, the Ministry of Health and Family Welfare (MOHFW) in collaboration with the Scaling Up of Zinc in Early Childhood (SUZY) team developed two committees: A National Advisory Committee, headed by the Health Secretary, and a Planning and Implementation Committee, headed by the Joint Secretary, Public Health and WHO. These committees acted as platforms for collaboration between policy-makers and researchers, facilitating the sharing of tacit knowledge and policy positions and the setting of common priorities and goals. For example, the committee suggested involving the Bangladesh Pediatric Association for their technical opinion as well as the Directorate General of Health Services (DGHS) to expedite the scaling up process [23].

Based on the evidence, the National Advisory Committee approved the policy on using zinc in addition to ORS for under-five children suffering from diarrhea and incorporated zinc into a revised National Diarrhea Treatment Guideline. Research also guided the development of the product, a dispersible zinc tablet, as well as its pricing. The following evidence-based policy changes were approved with regard to the national scale up of zinc in Bangladesh:

· Tablet formulation by the Bangladesh Drugs Administration,

· branding the product as “Baby Zinc”,

· over-the-counter sales waiver and

· mass media promotion of Baby Zinc.

An early misstep, trying to scale up with community health workers whose primary focus was family planning, led to the commissioning of qualitative studies to identify who should deliver the intervention. These studies discovered a cascade effect for adoption: Even though the product was available over-the-counter and could be easily administered, physicians and especially pediatricians were identified as key players in promoting and prescribing it [23].

Based on this evidence, the project embarked on a training blitz of all medical colleges and public health physicians at the district and sub-district level, as well as 8000 village doctors in their role as trainers for the more than 200,000 informal providers. Repeat impact surveys were conducted every three months and then annually in order to monitor for intended and unintended consequences. Rapid increase in zinc awareness occurred, from near zero prior to the launch to nearly 90% of urban and over 70% of rural caregivers. However, use of zinc lags far behind awareness in all settings with a national average of 17%. Within the rural poor and in urban slums coverage rates stagnated by the end of the first year of the campaign. Implementation research suggested that it was the lack of a recommendation from the provider that kept caretakers from using zinc for the first time. In addition to more targeted training at formal and informal providers and drug vendors, further research was undertaken to identify barriers to the sale of zinc among drug vendors [23,26].

The experience of scaling-up zinc use in Bangladesh reflects the factors identified by Ssengooba *et al.* (2011) as facilitating policy uptake and continued use of implementation research, they include:

· Shared platforms for learning and decision making,

· pilots to assess feasibility of interventions,

· evolution of agencies to undertake operational research and

· visibility of the benefits of the intervention [14].

## Strengthening implementation research and KT capacity in low and middle income countries

The KT process is often viewed in an oversimplified and linear fashion, built upon a singular input of research on the effectiveness of a health intervention, and not addressing the policy process as a whole, nor the capacity that organizations have for policy [27]. How can we encourage the use of KT and implementation research at both the organizational and country level? Although this article has provided reasoning for the generation of implementation research and its potential use in KT, preparing the policy environment to reap these benefits can be difficult. There must be sufficient capacity at the organizational and country levels to acquire, analyze, adapt and apply implementation research. While still rare [16], policy-maker involvement in KT is now being promoted by global initiatives such as EVIPNet, as well as regional and country initiatives [28,29].

There are many strategies for strengthening capacity to encourage evidence informed decision-making [16,30]. One systematic review looked at the barriers to and facilitators of evidence-informed decision-making in public health and, although focused in a high-income country, made the following recommendations that can be applied elsewhere:

· Encourage strong relationships between policy-makers and researchers as interactions increase the likelihood of research use by policy-makers,

· manage conflicts which arise between policy-makers and researchers,

· promote interactions between stakeholders and researchers and policy-makers so that decisions will be informed in part by stakeholder input,

· encourage collaboration between healthcare organizations and networks, particularly between newer organizations and more mature ones and

· encourage capacity building for research use among policy-makers as an effective strategy for research use [31].

One way to combine these strategies and create a fertile KT environment is to promote country mechanisms or KT platforms to systematically use evidence in policy-making in low and middle-income countries. EVIPNet is a successful example, a WHO programme with characteristics of a global social network that encompasses 26 KT platforms, also known as country teams. The paramount goal of each country team is to promote evidence informed decision-making in public health at national and other jurisdictional levels. In order to build capacity it is vital to continuously engage various participants in the policy-making process. As such, each country team consists of researchers, high-level decision-makers and other stakeholders (e.g. patients, healthcare workers and civil society representatives). The diversity of membership promotes sustainable partnerships between individuals and organizations and allows for the sharing of best practices and feedback.

EVIPNet holds capacity building workshops to enhance the knowledge translation capacity of policy-makers, researchers and other stakeholders. The diversity of stakeholders at workshops is intended to enhance the educational experience of attendees; EVIPNet promotes the philosophy of learning by doing together, so as to better work together. These capacity strengthening programmes place emphasis on producing tangible objects such as evidence-informed policy briefs, as well as the preparation of processes such as deliberative dialogues. This combined approach enables policy-makers to develop skills related to problem identification, framing a research problem, context mapping and priority setting, to name a few. The skills and lessons developed by policy-makers and researchers contribute to sustainable health systems strengthening.

The EVIPNet team in Burkina Faso’s work on access to artemisinin-based combination therapies (ACT) for uncomplicated malaria illustrates EVIPNet’s potential to strengthen knowledge translation capacity towards programme scale-up. The Burkina Faso’s EVIPNet team, consisting of policy-makers and researchers, prepared a policy brief that presented three viable policy options for supporting the widespread use of ACT to treat uncomplicated malaria:

· Engaging the private sector in adhering to national guidelines about subsidized drugs in all settings,

· motivating and retaining community health workers involved in the home management of malaria and

· banning monotherapies after ensuring the ACT is fully deployed across the country and that pharmacies are informed about the policy.

The policy brief was a key input to a national policy dialogue, involving senior government officials and key stakeholders, held to discuss how both the public and private sector can best support the widespread use of ACT. The policy brief also directly informed Burkina Faso’s successful application to the Global Fund for HIV-AIDS, TB and Malaria (GFATM) in its 7th Round.

The EVIPNet team’s work has now led to the implementation of the community health worker option through a pilot in three districts of the country, with the goal of a full-scale implementation by the 8th Round of the GFATM. An implementation research protocol (mostly a rapid ethnographic assessment) has been applied to each participating district, in order to monitor and evaluate the advantages, disadvantages, costs, barriers and facilitators in the execution of the policy option at the very specific district level. The other two options proposed in the policy brief are also being implemented through additional activities [32].

## Conclusions

Implementation research is an integral part of the KT continuum. Emphasis must be placed not only on its production, but also on its quality, proper use and uptake in decision-making. In order to more effectively implement evidence informed policy, policy-makers and researchers should learn together and work in partnership to improve access and delivery. Steps should be taken to increase the demand for research use and KT through sustainable partnerships and mechanisms, including KT platforms (at the district, provincial and national levels) that promote the early involvement of policy-makers, managers, health care providers and patients and serve as the basis for capacity-strengthening activities.

## Abbreviations

ACT, Artemisinin-based Combination Therapy; CIHR, Canadian Institutes for Health Research; DGHS, Directorate General of Health Services; EVIPNet, Evidence Informed Policy Network; GFATM, Global Fund for HIV-AIDS, TB and Malaria; KT, Knowledge Translation; MDG, Millennium Development Goals; MOHFW, Ministry of Health and Family Welfare; SUZY Project, Scaling Up of Zinc in Early Childhood Project.

## Competing interests

The authors report no competing interest.

## Authors’ contributions

UP: Designed the study, developed the outline, and contributed to the analysis, writing and revision of the report. TPK: Revised the outline, contributed to the analysis, writing and revision of the report, and wrote a case study. AA: Revised and reorganized the outline, contributed to the analysis, writing and revision of the report, and reviewed bibliography. PS: Developed the outline, contributed to the analysis, writing and revision of the report. TP: Developed the outline, contributed to the analysis, writing and revision of the report. JKK: Developed the outline, contributed to the analysis, writing and revision of the report. BM: Helped develop the outline and first manuscript, contributed to the analysis, writing and revision of the report. All authors read and approved the final manuscript.

## Authors’ information

The authors do not wish to disclose any additional professional information.
